# Genetic Ablation of Soluble TNF Does Not Affect Lesion Size and Functional Recovery after Moderate Spinal Cord Injury in Mice

**DOI:** 10.1155/2016/2684098

**Published:** 2016-12-14

**Authors:** Ditte Gry Ellman, Matilda Degn, Minna Christiansen Lund, Bettina Hjelm Clausen, Hans Gram Novrup, Simon Bertram Flæng, Louise Helskov Jørgensen, Lujitha Suntharalingam, Åsa Fex Svenningsen, Roberta Brambilla, Kate Lykke Lambertsen

**Affiliations:** ^1^Neurobiology Research, Institute of Molecular Medicine, J.B. Winsloewsvej 21, st, 5000 Odense C, Denmark; ^2^Department of Diagnostics, Molecular Sleep Lab, Rigshospitalet, Nordre Ringvej 69, 2600 Glostrup, Denmark; ^3^Department of Pathology, Department of Clinical Research, SDU Muscle Research Cluster, University of Southern Denmark, J.B. Winsloewsvej 15, 5000 Odense C, Denmark; ^4^The Miami Project to Cure Paralysis, University of Miami Miller School of Medicine, 1095 NW 14th Terrace, Miami, FL 33136, USA; ^5^Department of Neurology, Odense University Hospital, J.B. Winsloewsvej 4, 5000 Odense C, Denmark; ^6^Brain Research-Inter-Disciplinary Guided Excellence (BRIDGE), Department of Clinical Research, 5000 Odense C, Denmark

## Abstract

Traumatic spinal cord injury (SCI) is followed by an instant increase in expression of the microglial-derived proinflammatory cytokine tumor necrosis factor (TNF) within the lesioned cord. TNF exists both as membrane-anchored TNF (mTNF) and as cleaved soluble TNF (solTNF). We previously demonstrated that epidural administration of a dominant-negative inhibitor of solTNF, XPro1595, to the contused spinal cord resulted in changes in Iba1 protein expression in microglia/macrophages, decreased lesion volume, and improved locomotor function. Here, we extend our studies using mice expressing mTNF, but no solTNF (mTNF^Δ/Δ^), to study the effect of genetic ablation of solTNF on SCI. We demonstrate that TNF levels were significantly decreased within the lesioned spinal cord 3 days after SCI in mTNF^Δ/Δ^ mice compared to littermates. This decrease did, however, not translate into significant changes in other pro- and anti-inflammatory cytokines (IL-10, IL-1*β*, IL-6, IL-5, IL-2, CXCL1, CCL2, or CCL5), despite a tendency towards increased IL-10 and decreased IL-1*β*, TNFR1, and TNFR2 levels in mTNF^Δ/Δ^ mice. In addition, microglial and leukocyte infiltration, activation state (Iba1, CD11b, CD11c, CD45, and MHCII), lesion size, and functional outcome after moderate SCI were comparable between genotypes. Collectively, our data demonstrate that genetic ablation of solTNF does not significantly modulate postlesion outcome after SCI.

## 1. Introduction

Traumatic spinal cord injury (SCI) is most often caused by a sudden contusion of the spinal cord, where the initial tissue damage is followed by a second phase of cell death, inflammation, and degeneration that occurs over weeks and months after the initial trauma.

The microglial-derived cytokine tumor necrosis factor (TNF) increases at the lesion site within the first 1-2 hours after SCI in rodents [1–4] and spinal cord injured individuals display elevated TNF serum concentrations [5, 6], suggesting that TNF plays a major part in the development of secondary tissue damage [7].

TNF exists in two biologically active forms, a membrane-anchored form (mTNF) and a soluble form (solTNF), which is shed from the membrane by the metalloproteinase TNF-*α* converting enzyme (TACE/ADAM17). The biological effects of solTNF and mTNF are mediated through binding of TNF receptor 1 (TNFR1) and TNFR2, which differ in expression, ligand affinity, cytoplasmic tail structure, and downstream signaling pathways (reviewed in Probert [8]).

In experimental focal cerebral ischemia, an acute CNS injury, microglial-derived mTNF has been shown to be neuroprotective through binding to TNFR1 [9–12]. However, in SCI the data are conflicting. Kim and colleagues found TNFR1^−/−^ mice to have increased lesion size and worse functional outcome compared to controls [13], suggesting a protective role for TNFR1, whereas Genovese et al. [14] demonstrated reduced tissue damage and improved motor function in mice treated with the nonselective TNF inhibitor infliximab as well as in TNFR1^−/−^ mice, indicating a detrimental role for TNFR1. Surprisingly, germ-line ablation of TNF in TNF^−/−^ mice did not result in any differences in lesion size and functional outcome following SCI compared to controls [15]. We recently demonstrated that epidural administration of the dominant-negative solTNF inhibitor XPro1595 reduced lesion size and improved functional outcome following SCI, whereas etanercept, inhibitor of both mTNF and solTNF, had no effect [16]. Importantly, systemic administration of either compound was ineffective [16], in line with other studies showing that systemic administration of etanercept following SCI in mice does not reduce inflammation and tissue injury or infiltration of neutrophils nor improves the functional outcome [17]. Late blockage of peripheral TNF with etanercept was also ineffective in improving locomotor function in mice with SCI [18], while in rats it reduced tissue damage, improved hindlimb function, and facilitated myelin regeneration [19]. It should also be mentioned that a case report of a spinal cord injured patient treated chronically with etanercept for ankylosing spondylitis demonstrated reduced inflammation, reduced perilesional area, and improved motor recovery [20].

Even though the studies into the role of microglial-derived TNF following SCI are inconclusive, they clearly demonstrate that the TNF-TNFR signaling cascade plays an important part in tissue inflammation, although the contribution of solTNF versus mTNF to tissue damage and functional recovery remains to be elucidated. In this study, we investigated the effect of solTNF and mTNF in SCI using genetically modified mTNF^Δ/Δ^ mice that express only mTNF [21]. We show that absence of solTNF in mTNF^Δ/Δ^ mice does not affect lesion size and functional outcome 35 days after SCI. However TNF levels are significantly decreased within the lesioned spinal cord 3 days after SCI compared to littermate control mice (mTNF^wt/wt^). These findings suggest that genetic ablation of solTNF does not affect lesion size and functional outcome after SCI.

## 2. Materials and Methods

### 2.1. Mice

Homozygous mTNF^Δ/Δ^ and mTNF^wt/wt^ littermates were obtained by crossing heterozygous mTNF^Δ/wt^ mice at the Biomedical Laboratory, University of Southern Denmark (SDU) [12, 21]. These mice were originally generated by replacing the endogenous TNF allele with Δ1–9, K11E TNF allele [21]. This resulted in loss of TACE-mediated cleavage preventing shedding of solTNF [21, 22] but maintenance of normal cell-surface expression of mTNF [21]. All experiments were performed blinded on age-matched (8–12 weeks) female mTNF^Δ/Δ^ and mTNF^wt/wt^ littermates. Animals were housed in ventilated cages with 1–3 cage-mates at a 12 h light/dark cycle, under controlled temperature and humidity, and with free access to food and water.

Mice were cared for in accordance with the protocols and guidelines approved by The Danish Animal Inspectorate under the Ministry of Food and Agriculture (J. numbers 2008-561-1523 and 2013-15-2934-00924); experiments are reported in accordance with the ARRIVE guidelines, and all efforts were made to minimize pain and distress.

### 2.2. Genotyping

DNA was extracted from tail biopsies from 3-4-week-old mice using a NucleoSpin Tissue kit (Macherey-Nagel) according to the manufacturer's instructions. DNA was amplified by PCR under the following conditions: 50°C for 2 minutes and 95°C for 10 minutes followed by 39 cycles of 95°C for 15 sec, 62°C for 1 minute, and 72°C for 1 minute and the following per PCR reaction: 12.5 *µ*L Master Mix (Thermo Scientific), 1 *µ*L forward primer (5′-GCGTCCAGCTGACTAAA), 1 *µ*L reverse primer (3′-ACCACTAGTTGGTTGCTTTGAGAT), and 10 *µ*L dH_2_O. Both primers were from DNA Technology A/S and in working concentrations of 10 pmol/*µ*L. The PCR products were visualized using the FlashGel system (Lonza). Four-microliter PCR products mixed with FlashGel loading buffer (Lonza) were loaded on FlashGel DNA cassette 1.2% agarose gel with 16 + 1 double wells (Lonza) and the gels were allowed to run for 25–30 minutes at 70 volts. In all gels, FlashGel system DNA marker (Lonza) and samples with known genotypes (homozygote, heterozygote, and wild type) were included.

### 2.3. Behavioral Analysis

We have previously performed a thorough behavioral assessment of mTNF^Δ/Δ^ in naïve conditions and found no differences in behavioral phenotype compared to mTNF^wt/wt^ littermates [12].

*Elevated Plus Maze*. To further examine anxiety-like behavior and locomotion, which could affect locomotor function after SCI, naïve mice were subjected to the elevated plus maze (EPM). The elevated plus maze apparatus consisted of two open arms and two closed arms (30 cm × 5 cm). The entire maze was elevated around 40 cm from the floor. Each mouse was placed in the center of the maze with the head facing towards the open arm. During a 5 min test, the time spent in the closed and open arms and the total distance moved were recorded using the SMART video tracking software (Panlab).

### 2.4. Contusive Spinal Cord Injury

Mice were anaesthetized using a ketamine (100 mg/kg, VEDCO Inc)/xylazine (10 mg/kg, VEDCO Inc) cocktail, laminectomized between vertebrae T8 and T10 and injured with an Infinite Horizon-0400 SCI Contusion Devise (Precision Systems and Instrumentation, LLC) by lowering the impactor onto the exposed cord at an impact of 75 Kdynes resulting in approximately 500 *μ*m displacement (moderate injury) [16]. Mice were then sutured, injected with saline to prevent dehydration, and given buprenorphine hydrochloride (0.001 mg/20 g body weight Temgesic) four times at 8-hour intervals for postsurgical analgesia. Mice were housed separately in a recovery room and monitored for a 24- to 48-hour recovery period. Thereafter, mice were observed twice daily for activity level, respiratory rate, and general physical condition. Manual bladder expression was performed twice a day until bladder function was regained. Body weight was monitored weekly. In addition, mice received s.c. prophylactic injections of antibiotic gentamicin (40 mg/kg) for 7 days to prevent urinary tract infections. The persons performing the SCI have attended the SCI Research Training Program at the Ohio State University. In total, 2 mTNF^Δ/Δ^ mice died during surgery, while 1 mTNF^wt/wt^ and 1 mTNF^Δ/Δ^ mouse were euthanized on day 8 and day 24, respectively, due to poor general health status.

### 2.5. Assessment of Functional Outcome after SCI

*Basso Mouse Scale*. Functional recovery of hind limb function after SCI was determined by scoring of the locomotor hindlimb performance in the open field using the Basso Mouse Scale (BMS) system, a 0 to 9 rating system designed specifically for the mouse [16, 23]. Under observer-blinded conditions, mice were evaluated over a 4-min period 1 and 3 days after SCI and weekly thereafter. Only mice with a score below 2, representing a successful lesion, on day 1 were included in the study. Before surgery, mice were handled and pretrained in the open field to prevent fear and/or stress behaviors that could bias the locomotor assessment. 

*Thermal Hyperalgesia*. Thermal hyperalgesia (hind paw withdrawal from a normally innocuous heat source) was tested with a Hargreaves' heat source using the Plantar Test apparatus (Ugo Basile) [16]. Each paw was tested 5 times with at least 2 min break in between. The lowest and highest reflex latency scores of each paw were discarded and the bilateral mean was calculated and plotted. The behavioral test was performed weekly when the mice reached a BMS score of 5 and were capable of frequent or consistent stepping and thereby plantar placement of the paws, typically around 3 weeks after SCI.

*Rung Walk*. In order to test stepping, interlimb coordination, and balance, mice were tested on the rung walk also when they reached a BMS score of 5. The rung walk consisted of two plates of transparent polymer, approximately 110 cm × 20 cm, with a 2.5 cm space between them. The apparatus was placed on two cages with the home cage at one end, making the mice automatically walk in that direction. To avoid stopping or turning during trials, animals were pretrained 5 times prior to surgery with the final test serving as baseline. Following SCI, eligible mice were tested at 3, 4, and 5 weeks using a handheld GoPro HD camera with 48 fps. Data were evaluated frame by frame using QuickTime. Left and right scores were calculated as follows: 6, complete miss; 5, touching rung but sliding off and losing balance; 4, touch, miss but no loss of balance; 3, replacement, mouse placed paw on rung but quickly moves it; 2, recorrection, aims for a rung but changes direction; 1, anterior or posterior placement; 0, perfect step. The total number of mistakes was plotted for analysis as previously described [16].

*Open Field Test*. The open field test was performed with a nontransparent squared plastic box (45 × 45 × 45 cm) over a period of 10 min 35 days after SCI [16]. Movements were tracked using the SMART video tracking software (Panlab) connected to a video camera (SSC-D378P, Biosite). The distance travelled (m), speed (cm/sec), and entries into the three zones (wall, interperiphery, and center of the box) were recorded automatically. A center/perimeter ratio was calculated based on the number of entries. Rearing, grooming, jumping, digging, urination, and droppings were recorded manually and presented as number (*n*) of events [24].

### 2.6. Tissue Processing

#### 2.6.1. Histopathology and Immunohistochemistry

For paraffin histopathology and immunohistochemical analyses, mice were deeply anaesthetized using an overdose of pentobarbital (200 mg/mL) containing lidocaine (20 mg/mL) and perfused through the left ventricle with ice-cold 4% paraformaldehyde (PFA) in phosphate-buffered saline (PBS). The spinal cords were quickly removed and tissue segments containing the lesion area (1 cm centered on the lesion) were paraffin-embedded and cut into 10 parallel series of 15 *μ*m thick microtome sections. Sections were stored at room temperature until further processing.

#### 2.6.2. Klüver-Barrera Luxol Fast Blue Staining for Myelinated Fibers

For evaluation of lesion pathology, 1 series of sections from each animal was stained in Luxol Fast Blue (LFB) (0.1% LFB in 95% ethanol (EtOH) and 0.05% acetic acid) at 60°C over night. Next day, sections were rinsed in 96% EtOH and distilled H_2_O, immersed briefly in lithium carbonate (0.05% Li_2_CO_3_ in distilled water), and differentiated in 70% EtOH. Next, sections were rinsed thoroughly in distilled H_2_O and immersed in 0.05% lithium carbonate to stop further differentiation. Sections were then placed in hematoxylin, rinsed in running tap water, and immersed briefly in eosin solution. Finally, sections were rinsed in 70% EtOH, followed by 3x 99% EtOH, placed in 3x xylene prior to mounting with Depex. Prior to staining, paraffin embedded sections were deparaffinized 3x 3 min in xylene, 3x 2 min in 99% EtOH, and 2x 2 min in 96% EtOH.

#### 2.6.3. Immunohistochemical Staining for CD45, F4/80, and MBP

Heat-Induced Antigen Retrieval was done on paraffin embedded sections by boiling the sections in citrate buffer + 0.05% Tween, pH 6.0 (MBP), Tris-EGTA buffer, pH 9.0 (CD45), or TRS buffer (Target Retrieval Solution, Dako) (F4/80) first 15 min at 900 W and then 9 min at 440 W. The sections were allowed to cool in the buffer before they were blocked for endogenous peroxidase and biotin activity. Sections were then incubated with anti-CD45 IgG (30-F11 (Ly 5); BD Pharmingen) diluted 1 : 100, anti-F4/80 IgG (AbD Serotec) diluted 1 : 100, or anti-MBP IgG (Biolegend) diluted 1 : 150 and detected using biotin-labeled anti-rat (Dako), diluted 1 : 200, or anti-mouse, diluted 1 : 100, antibodies, followed by ready-to-use anti-rabbit horse-radish perioxidase- (HRP-) labeled polymer (EnVision+ System, Dako) with diaminobenzidine (DAB) as chromogen (Dako). Nuclei were counterstained using Mayer's haemalum w/4.5% chloralhydrate. As negative controls, staining specificity was tested on parallel sections of spinal cord tissue by omitting the primary antibody or substitution with a primary antibody with a similar concentration of a mouse IgG1 isotype control (Dako) (for MBP) in order to check for any unspecific reaction from the detection system. As positive control for antibody specificity, a mouse multiblock containing several different tissues, including lymphatic organs, was included. Activation patterns were investigated in 5 sections (representing 750 *μ*m spinal cord) centered on the lesion epicenter from each animal.

#### 2.6.4. Immunofluorescent Staining for Glial Fibrillary Acidic Protein (GFAP) and Ionized Calcium Binding Adaptor Molecule (Iba1)

One series of sections from each animal was deparaffinized and rehydrated by placing the sections 3x 3 minutes in xylene, 3x 2 minutes in 99% EtOH, 2x 2 minutes in 96% EtOH, 2 minutes in 70% EtOH, and finally 5 minutes in running tap water. The sections were then demasked with TEG-buffer by placing the sections in warm TEG-buffer in a steamer for 15 minutes and then letting them cool for 15 minutes at room temperature before rinsing them for 15 minutes in running tap water. The sections were then rinsed 3x 15 minutes in Tris-buffered saline (TBS) before they were preincubated with 10% fetal bovine serum (FBS) in TBS with 0.5% Triton X-100 for 30 minutes. Hereafter the sections were incubated with Alexa Fluor® 488-conjugated anti-GFAP IgG (mouse monoclonal, clone 131-17719, Thermo Fischer Scientific) diluted 1 : 400 or anti-Iba1 IgG (Rabbit, Dako) diluted 1 : 400 1 hour at room temperature and hereafter over night at 4°C.

At day 2, sections were placed at room temperature for 30 minutes before they were rinsed in TBS for 10 minutes and then in TBS with 0.1% Triton X-100 for 10 minutes. Sections were then either stained with NeuroTrace® 530/615 Red Fluorescent Nissl Stain (Thermo Fischer Scientific) for 20 minutes (GFAP) or incubated with Alexa Fluor 488-conjugated chicken anti-rabbit IgG (Thermo Fischer Scientific) 1 : 500 for 2 hours and then rinsed 2 × 10 minutes in TBS before the nuclei were immersed in a TBS solution containing 10 *μ*M diamidino-2-phenylindole (DAPI) for 10 minutes. The sections were shortly rinsed in distilled water before they were mounted with ProLong Diamond. Activation patterns were investigated in 5 sections (representing 750 *μ*m spinal cord) centered on the lesion epicenter from each animal. Control reactions were performed by omitting the primary antibody or by substituting the primary antibody with Alexa Fluor 488 conjugated mouse IgG1_kappa_ (Thermo Fischer Scientific) or substituting the primary antibody with rabbit serum (Dako) diluted to the same concentration as the primary antibody. Sections were devoid of staining in the FITC imaging filter.

### 2.7. Lesion Volume Estimation

The volume of the lesion was determined from the area of every 10th LFB- or GFAP-stained section sampled by systematic uniform random sampling. The area of the lesion site was estimated in LFB-stained sections as previously described [16] using the VisioMorph software (Visiopharm) and the Cavalieri principle for volume estimation. For estimation of the lesion area in GFAP-stained sections, photomicrographs were acquired using an Olympus BX51 microscope with an Olympus DP73 camera connected to a PC set up with the Olympus CellSens software. Lesion size was then estimated using ImageJ analysis software (NIH) as per directions of the ImageJ developers (http://rsb.info.nih.gov/ij/). Analysis performed on digital images was carried out on unmanipulated pictures. On the presented pictures the contrast and curves have been adjusted to allow readers to appreciate the details on small-scale figures.

### 2.8. Estimation of White Matter

The area of intact white matter, based on MBP staining, was estimated as a percentage of the total spinal cord tissue area. Estimations were based on 5 sections centered on the epicenter and 5 sections located 300 *μ*m rostral to the lesion (*n* = 10 sections from 2-3 animals/group).

### 2.9. Flow Cytometry

#### 2.9.1. Isolation of Cells for Flow Cytometry

Mice were perfused with PBS as described above, the spinal cords were quickly removed, and tissue segments containing the lesion area (2.5 cm centered on the lesion) and perilesion area (0.5 cm distal to and 0.5 cm proximal to the lesion were pooled to represent perilesion tissue) were placed in cold Hank's Buffered Salt Solution (HBSS). Tissue from individual mice was processed separately. Single-cell suspensions were obtained by homogenization in HBSS using 70-*µ*m nylon cell strainers (BD Falcon). Cells suspensions were centrifuged at 300 ×g for 10 minutes at 4°C. The pellet was resuspended in PBS containing 0.5% FBS and Myelin Removal Beads II (Miltenyi Biotec) were added and the suspensions were incubated for 15 minutes at 4°C. Thereafter PBS containing 0.5% FBS was added and the suspensions were centrifuged at 300 ×g for 10 minutes at 4°C, after which the pellet was resuspended in PBS containing 0.5% FBS. The LS column (Miltenyi Biotec) was placed in the magnetic field of the MACS separator (Miltenyi Biotec) and columns prepared by washing with PBS containing 0.5% FBS. Next, the cell suspension was added and the flow-through was collected and centrifuged at 300 ×g for 10 minutes at 4°C. The pellet was washed by resuspending in PBS and centrifuged at 300 ×g for 10 minutes at 4°C. To lyse the red blood cells the pellet was resuspended in 0.83% ammonium chloride for 10 minutes after which the suspension was centrifuged at 300 ×g for 10 minutes at 4°C. The pellet was washed by resuspending in PBS and centrifuged at 300 ×g for 10 minutes at 4°C. Finally the pellet was resuspended in PBS and the cell suspension was stained for live/dead cells using Fixable Viability Dye eFlouro 506 (eBioscience) for 20 minutes at 4°C in the dark. The pellet was washed by resuspending in PBS and centrifuged at 300 ×g for 10 minutes at 4°C. Cells were fixed in Cytofix/Cytoperm (BD Pharmingen) for 15 minutes at 4°C and centrifuged at 300 ×g for 10 minutes at 4°C. The pellet was washed twice in FACS staining buffer (HBSS containing 2% FBS and 0.1% sodium azide) and centrifuged at 300 ×g for 10 minutes at 4°C in between. Finally the pellet was resuspended in FACS staining buffer and kept in the dark at 4°C until staining for flow cytometry.

#### 2.9.2. Flow Cytometry

The cells were blocked in 10% rat serum for 30 minutes on ice before they were washed with PBS and centrifuged at 600 ×g for 5 minutes at 4°C. The cells were resuspended in FACS staining buffer and stained with combinations of PE-CD11b (BD Biosciences, clone M1/70), PerCPCy5.5-CD45 (BD Biosciences, clone 30-F11), APC-CD3 (BD Biosciences, clone 145-2C11), PE-Cy7-Gr1 (Biolegend, clone RB6-8C5), PE-Cy7-CD11c (BD Biosciences, clone HL3), and 647-MHC class II (BD Biosciences, clone M5/114.15.2) antibodies for 30 minutes on ice. Finally, the cells were washed in PBS, centrifuged at 600 ×g for 5 minutes at 4°C, and resuspended in FACS buffer before they were run on a FACSverse flow cytometer, and 10^6^ events were acquired per sample using forward scatter (FSC) and side scatter (SSC). The analysis was performed using the FACSuite software [25]. Positive staining was determined based on the respective isotype controls and the respective fluorescent minus one (FMO) controls. The mean fluorescent intensity (MFI) was calculated as the geometric mean of each population in the CD45 and CD11b gates, respectively [25].

### 2.10. Multiplex and Western Blotting Analyses

*Protein Purification*. Whole spinal cord protein samples from mTNF^Δ/Δ^ and mTNF^wt/wt^ mice exposed to SCI and allowed 3-day survival in addition to naïve mTNF^Δ/Δ^ and mTNF^wt/wt^ mice were obtained and protein extractions were prepared as previously described [11, 12].

*Multiplex Analysis*. To measure cytokine, chemokine, and TNFR protein levels by the MSD Mouse Proinflammatory V-Plex Plus Kit (IFN*γ*, IL-1*β*, IL-2, IL-4, IL-5, IL-6, IL-10, IL-12p70, CXCL1, and TNF; K15012C, Mesoscale) and mouse TNF-RI, TNF-RII, RANTES, and MCP-1 Ultra-Sensitive Kits (Mesoscale), we used a SECTOR Imager 6000 (Mesoscale Discovery) Plate Reader according to the manufacturer's instructions. Samples were diluted twofold in Diluent 41 prior to measurement and samples were run in duplicate [11]. Data was analyzed using MSD Discovery Workbench software.

*Western Blotting Analysis*. Western blot analysis for Iba1 (1 : 500, Wako) was performed using 18 *μ*g protein extract separated on 4–12% SDS-PAGE gels (Invitrogen) using MOPS SDS (Invitrogen) containing 0.25% antioxidant (Invitrogen) essentially as previously described [11]. *α*-Actin (1 : 100,000, Millipore) was used as loading control. SeeBlue prestained standard (Invitrogen) was used as a molecular weight marker. Bands were quantified using Image Lab Software (Bio-Rad). Analysis was performed with *n* = 4 mice/group and data were normalized to *α*-actin and presented as percentages relative to mTNF^wt/wt^ mice.

As a control, 2 independent gels were prepared where the primary antibody was omitted in the protocol. Development of the membrane showed the absence of a 17 kDa band, corresponding to the size of Iba1.

### 2.11. Statistical Analysis

Comparisons were performed using repeated measures (RM) two-way ANOVA followed by multiple *t*-test analysis (BMS, rung walk, Hargreaves' tests, and weight change), Mann–Whitney, or Wilcoxon matched-pairs signed rank tests. Analyses were performed using Prism 4.0b software for Macintosh (GraphPad Software). Statistical significance was established for *p* ≤ 0.05.

## 3. Results

### 3.1. Genetic Ablation of solTNF Does Not Alter Locomotor Function or Anxiety-Related Behavior

**Figure 1:**
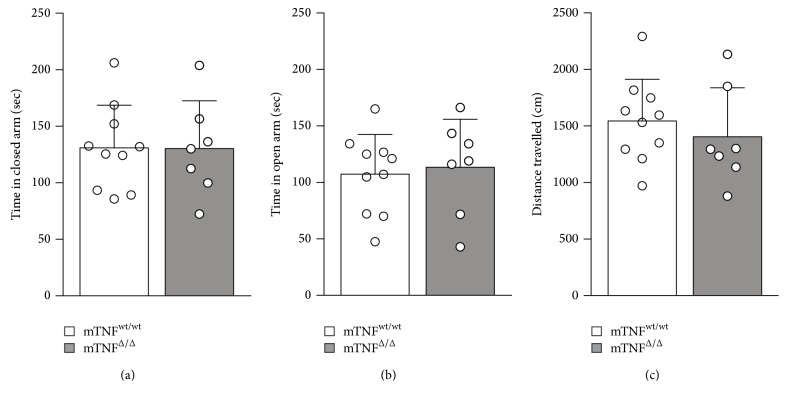
Elevated plus maze analysis in naïve mTNF^wt/wt^ and mTNF^Δ/Δ^ mice. (a–c) mTNF^wt/wt^ and mTNF^Δ/Δ^ mice spent comparable times in the closed arm (a) and the open arm (b) and travelled a similar distance (c) in the elevated plus maze test. Mann–Whitney test, *n* = 7–10 mice/group. Results are presented as mean ± SD.

Using the open field, rotarod, and Y-maze tests, we previously demonstrated that mTNF^Δ/Δ^ mice display no obvious behavioral abnormalities [12]. This was further validated in the present study using the EPM test. We found that the time spent in the closed (Figure 1(a)) and open arms (Figure 1(b)) and the total distance travelled in the EPM were comparable between mTNF^wt/wt^ and mTNF^Δ/Δ^ mice (Figure 1(c)). Together our findings demonstrate that locomotor function following SCI will not be affected by innate changes in the behavioral phenotype between naïve mTNF^wt/wt^ and mTNF^Δ/Δ^ mice.

### 3.2. Genetic Ablation of solTNF Does Not Affect Lesion Size or Functional Outcome after SCI

**Figure 2:**
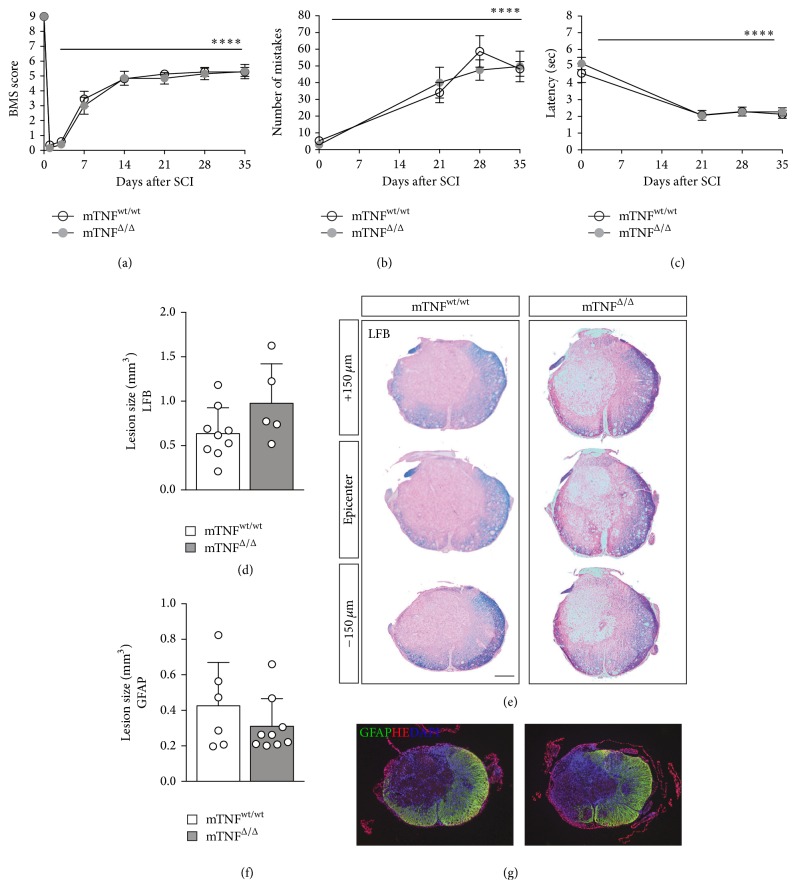
Genetic ablation of solTNF does not affect functional outcome or lesion size after SCI. (a) Analysis of BMS scores in mTNF^wt/wt^ and mTNF^Δ/Δ^ mice showed that genetic ablation of solTNF did not affect BMS scores after SCI. Both groups of mice improved their BMS score over time (two-way RM ANOVA; time ^*∗∗∗∗*^ *p* < 0.0001,  *F*_3,133_ = 295.1), *n* = 10-11 mice/group. (b) Rung walk analysis showed that both groups of mice increased their number of mistakes after SCI (two-way RM ANOVA; time ^*∗∗∗∗*^ *p* < 0.0001,  *F*_3,51_ = 27.8, Tukey's* post hoc* ^*∗∗∗∗*^ *p* < 0.0001), *n* = 9-10 mice/group, and no differences between genotypes were observed. (c) Thermal stimulation using Hargreaves' test showed no differences in latency time to withdraw paws between genotypes. Both groups decreased latency to remove their paws over time after SCI (two-way RM ANOVA; time ^*∗∗∗∗*^ *p* < 0.0001,  *F*_3,51_ = 14.72, Tukey's* post hoc* ^*∗∗∗∗*^ *p* < 0.0001), *n* = 9-10 mice/group. (d) Analysis of lesion volumes in Luxol Fast Blue (LFB) stained sections 35 days after SCI showed that lesion size was comparable between mTNF^wt/wt^ and mTNF^Δ/Δ^ mice (Mann–Whitney), *n* = 5–9 mice/group. (e) Representative LFB-stained thoracic spinal cord sections from mTNF^wt/wt^ and mTNF^Δ/Δ^ mice allowed 35-day survival after SCI. Scale bar: 100 *μ*m. (f) Analysis of lesion volumes in GFAP-stained sections 35 days after SCI showed that lesion size was comparable between mTNF^wt/wt^ and mTNF^Δ/Δ^ mice (Mann–Whitney), *n* = 5–9 mice/group. (g) GFAP labeling (green), fluorescent Nissl (red), and DAPI (blue) staining of representative spinal cord sections from mTNF^wt/wt^ and mTNF^Δ/Δ^ mice allowed 35-day survival after SCI. Results are presented as mean ± SD.

On the basis of our previous findings of a neuroprotective effect of mTNF [12, 16], we investigated whether genetic ablation of solTNF in mTNF^Δ/Δ^ mice could also improve functional recovery and reduce tissue damage following traumatic SCI. In this study, mTNF^wt/wt^ and mTNF^Δ/Δ^ mice were subjected to SCI and locomotor performance in the open field was recorded on days 1 and 3 and then weekly for 5 weeks and scored with the BMS. We found that there were no differences in BMS scores between the two genotypes at any time point after SCI (final BMS ± SEM; mTNF^wt/wt^: 5.3 ± 1.0; mTNF^Δ/Δ^: 5.3 ± 1.5) (Figure 2(a)). Both genotypes improved their BMS scores significantly over time. Mice were also evaluated using rung walk analysis when they reached a BMS score of 5 and weekly thereafter. Even though both genotypes displayed significant changes over time, no differences were observed between mTNF^wt/wt^ and mTNF^Δ/Δ^ mice (Figure 2(b)). Thermal hyperalgesia was tested using Hargreaves' test. Despite findings of significant changes in nociception over time in both mTNF^wt/wt^ and mTNF^Δ/Δ^ mice, no difference between genotypes was observed (Figure 2(c)). Finally in order to investigate the effect of genetic ablation of solTNF on general activity and anxiety-related behavior after SCI, mTNF^wt/wt^ and mTNF^Δ/Δ^ mice were subjected to the open field test 35 days after SCI (Supplemental Figure 1, Supplementary Material available online at http://dx.doi.org/10.1155/2016/2684098). We found no differences in the total distance travelled (Supplemental Figure 1A), in the speed at which the mice moved (Supplemental Figure 1B), in number of rearings (Supplemental Figure 1C), nor in number of zone changes (*p* < 0.05, mTNF^wt/wt^: 45.0 ± 33.9; mTNF^Δ/Δ^: 37.5 ± 31.2), demonstrating that mTNF is sufficient to sustain locomotor function. Also, grooming (Supplemental Figure 1D) and urination (Supplemental Figure 1E) and the center/perimeter ratio (*p* < 0.05, ratios: mTNF^wt/wt^: 0.08 ± 0.09%; mTNF^Δ/Δ^: 0.06%  ± 0.10) were comparable between mTNF^wt/wt^ and mTNF^Δ/Δ^ mice, and only the number of droppings differed significantly between the two genotypes (Supplemental Figure 1F). In line with these data, we observed no difference in lesion size (Figures 2(d)–2(f)) or the percentage of MBP^+^ white matter area (mTNF^wt/wt^: 48.6%  ± 10.3%; mTNF^Δ/Δ^: 49.4%  ± 12.0%) between mTNF^wt/wt^ and mTNF^Δ/Δ^ mice 35 days after SCI. Intense GFAP immunoreactivity was detected both in genotypes around the lesion and in the surrounding white matter, indicating formation of a glial scar (Figure 2(g)).

Body weight was affected over time by SCI, but there was no difference between genotypes (Supplemental Figure 1G).

### 3.3. Genetic Ablation of solTNF Reduces TNF Levels after SCI

**Figure 3:**
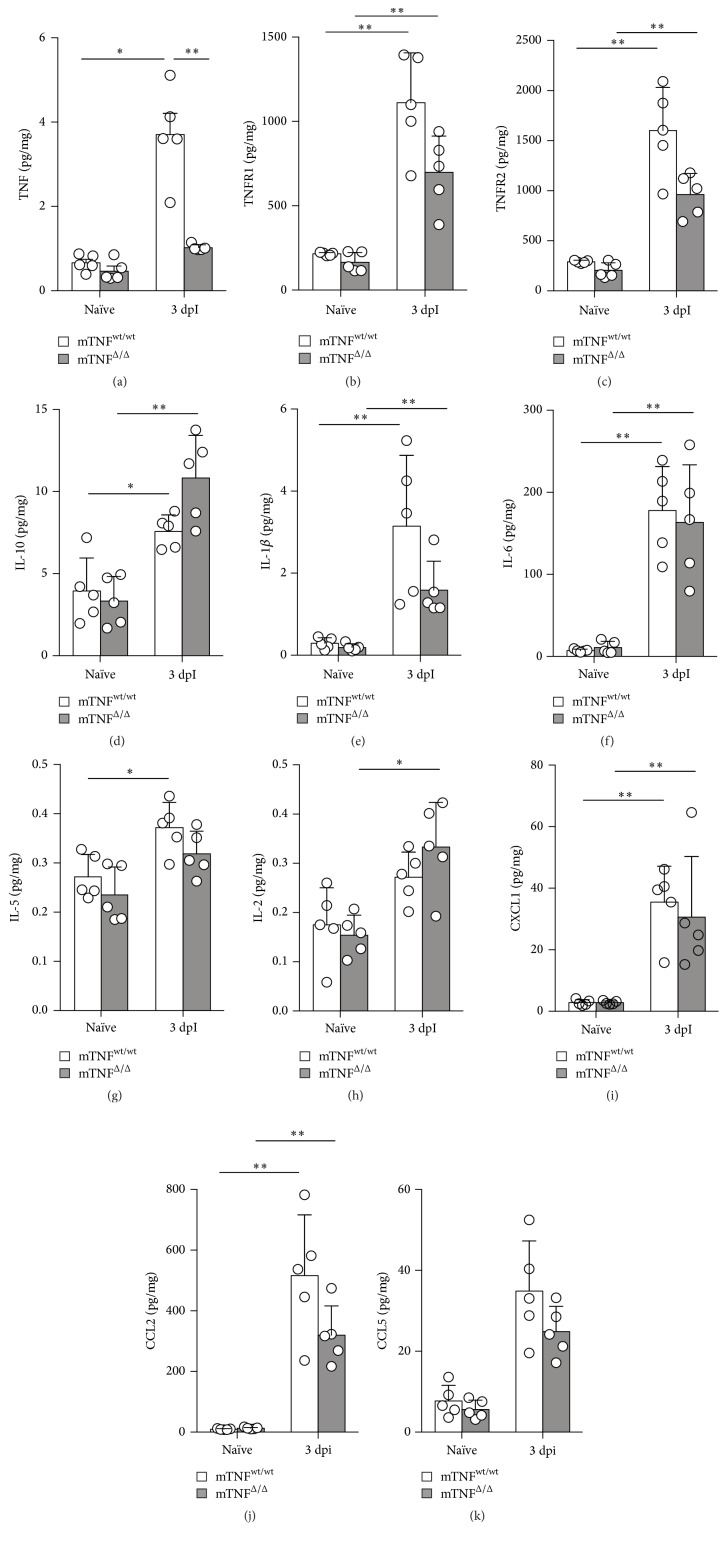
Cytokine and TNFR expression profiling after SCI. (a–k) TNF (a), TNFR1 (b), TNFR2 (c), IL-10 (d), IL-1*β* (e), IL-6 (f), IL-5 (g), IL-2 (h), CXCL1 (i), CCL2 (j), and CCL5 (k) protein levels were quantified by multiplex technology in naïve mice and 3 days after SCI in mTNF^wt/wt^ and mTNF^Δ/Δ^ mice. For each protein, results are expressed as mean ± SD, *n* = 5 mice/group. ^*∗*^ *p* < 0.05 and ^*∗∗*^ *p* < 0.01, Mann–Whitney test.

We previously showed that TNF is decreased in mTNF^Δ/Δ^ mice compared to mTNF^wt/wt^ controls after experimental stroke [12]. Following SCI, the genetic ablation of solTNF also affected TNF in the spinal cord significantly (Figure 3(a)). TNF increased significantly in mTNF^wt/wt^ mice compared to naïve conditions and was significantly increased compared to mTNF^Δ/Δ^ mice 3 days after SCI, whereas TNF stayed at baseline levels after SCI in mTNF^Δ/Δ^ mice (Figure 3(a)).

TNFR1 (Figure 3(b)) and TNFR2 (Figure 3(c)) were also significantly affected in the spinal cords of mTNF^wt/wt^ and mTNF^Δ/Δ^ mice. We found that, in both mTNF^wt/wt^ and mTNF^Δ/Δ^ mice, TNFR1 and TNFR2 increased significantly in the lesioned spinal cord 3 days after SCI compared to naïve conditions but with no significant difference between genotypes (Figures 3(b) and 3(c)). However, for both TNFR1 and TNFR2 there was a tendency towards reduced TNFR1 (*p* = 0.06) and TNFR2 (*p* = 0.06) levels in mTNF^Δ/Δ^ compared to mTNF^wt/wt^ mice 3 days after SCI.

### 3.4. Genetic Ablation of mTNF Does Not Affect Neuroinflammation in the Lesioned Spinal Cord 3 Days after SCI

IL-10 (Figure 3(d)), IL-1*β* (Figure 3(e)), and IL-6 (Figure 3(f)) were significantly increased in mTNF^wt/wt^ and mTNF^Δ/Δ^ mice 3 days after SCI compared to naïve conditions, but with no significant difference between genotypes. However, there was a tendency towards increased IL-10 (*p* = 0.09) and decreased IL-1*β* (*p* = 0.09) in mTNF^Δ/Δ^ compared mTNF^wt/wt^ mice 3 days after SCI. IL-5 (Figure 3(g)) was significantly increased at 3 days compared to naïve conditions only in mTNF^wt/wt^ mice (Figure 3(h)), whereas IL-2 only increased significantly in mTNF^Δ/Δ^ mice (Figure 3(h)). IFN*γ* and IL-4 protein levels were not affected 3 days after SCI (not shown).

CXCL1 (Figure 3(i)) and CCL2 (Figure 3(j)) were significantly upregulated in the lesioned spinal cord of mTNF^wt/wt^ and mTNF^Δ/Δ^ mice 3 days after SCI compared to naïve conditions, whereas the increase in CCL5 (Figure 3(k)) did not reach statistical significance. No differences in CXCL1, CCL2, and CCL5 were observed between mTNF^wt/wt^ and mTNF^Δ/Δ^ mice (Figures 3(i)–3(k)).

### 3.5. Genetic Ablation of solTNF Does Not Affect Monocyte/Macrophage Infiltration into the Lesioned Spinal Cord 3 Days after SCI

**Figure 4:**
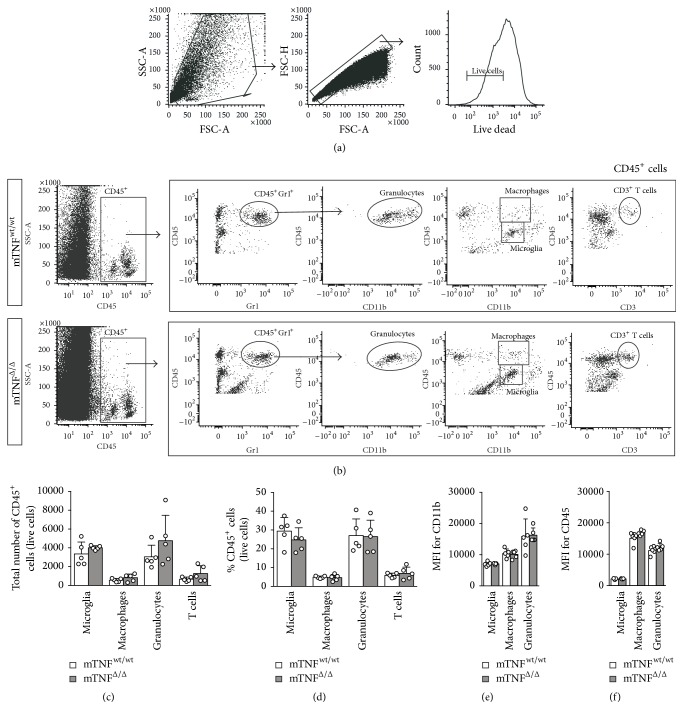
Flow cytometry of microglia and infiltrating leukocytes 3 days after SCI. (a) Gating strategy: FSC/SSC was used to define cell populations. Singlet cells were identified using FSC-A/FSC-H, and only live cells were included. (b) Representative flow cytometry plots comparing macrophage, granulocyte, and T cell infiltration in mTNF^wt/wt^ and mTNF^Δ/Δ^ mice. (c, d) Number (c) and percentage (d) of microglia (CD11b^+^CD45^dim^), macrophages (CD11b^+^CD45^dim^Gr1^−^), granulocytes (CD11b^+^CD45^dim^Gr1^+^), and T cells (CD45^+^CD3^+^) in mTNF^wt/wt^ and mTNF^Δ/Δ^ mice 3 days after SCI. Results are expressed as mean ± SD, *n* = 5 mice/group, Mann–Whitney test.

We have previously shown that monocyte/macrophage infiltration into the ischemic brain is decreased in mTNF^Δ/Δ^ mice 24 hours after focal cerebral ischemia compared to mTNF^wt/wt^ mice [12]. Based on this, we evaluated microglia (CD11b^+^CD45^dim^ cells), macrophage (CD11b^+^CD45^high^Gr1^−^), granulocyte (CD11b^+^CD45^high^Gr1^+^), and T cell (CD45^+^CD3^+^) populations 3 days after SCI using flow cytometry (Figures 4(a) and 4(b)) and found no differences in total numbers (Figure 4(c)) and percentages (Figure 4(d)) between mTNF^wt/wt^ and mTNF^Δ/Δ^ mice. As CD45 MFI values in microglia have been shown to be affected by XPro1595 and etanercept treatment following experimental stroke, indicating increased activation by these cells [25], we also investigated CD11b and CD45 MFI levels in microglia, macrophages, and granulocytes 3 days after SCI (Figures 4(e) and 4(f)). However, no difference between genotypes was observed in MFI levels for either CD11b (Figure 4(e)) or CD45 (Figure 4(f)) in any of the cell populations investigated.

### 3.6. Genetic Ablation of solTNF Does Not Affect the Activation State of Microglia and Leukocytes 3 and 35 Days after SCI

**Figure 5:**
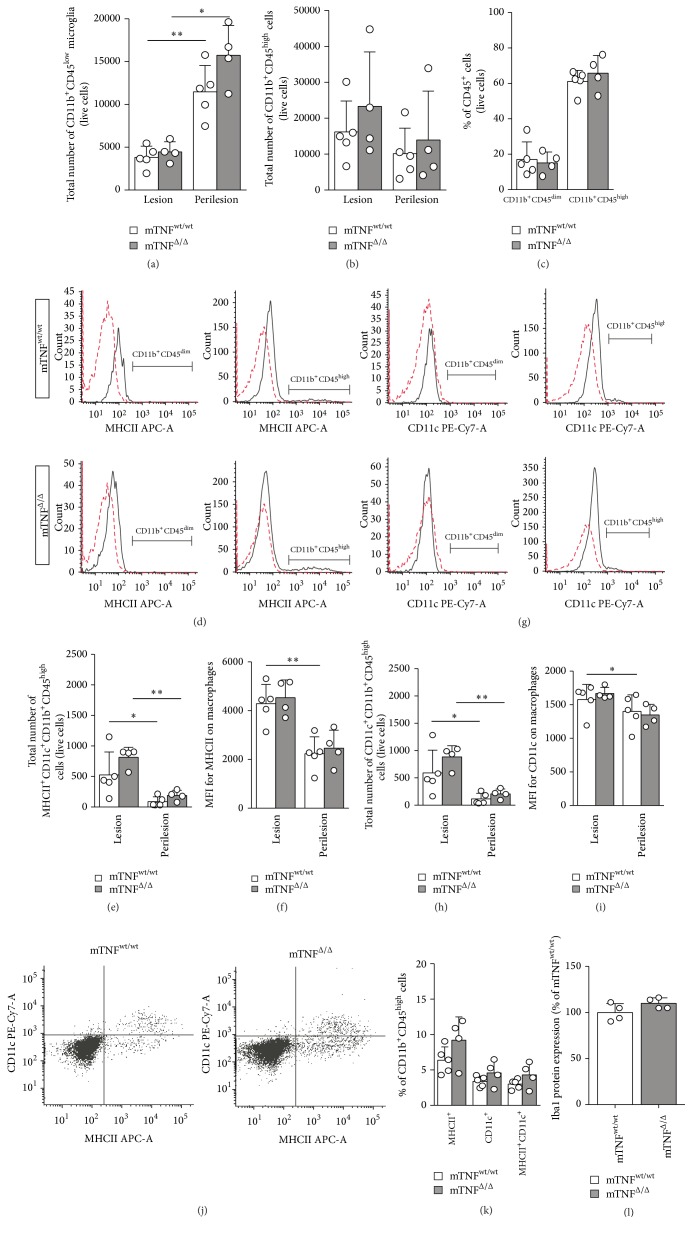
Flow cytometric analysis of the activation state of microglia and infiltrating cells 3 days after SCI. (a–c) Number of CD11b^+^CD45^dim^ microglia (a) and CD11b^+^CD45^high^ cells (b) and percentages of microglia and infiltrating cells (c) within the lesion and in the perilesion area 3 days after SCI. (d) Representative histograms of MHCII expression on CD11b^+^CD45^low^ microglia and CD11b^+^CD45^high^ cells located in the lesions of mTNF^wt/wt^ and mTNF^Δ/Δ^ mice 3 days after SCI. (e, f) The number of MHCII^+^CD11b^+^CD45^high^ cells (e) and MFI for MHCII (f) were comparable between mTNF^wt/wt^ and mTNF^Δ/Δ^ mice. (g) Representative histograms of CD11c expression on microglia and infiltrating cells located in the lesions of mTNF^wt/wt^ and mTNF^Δ/Δ^ mice 3 days after SCI. (h, i) The number of CD11c^+^CD11b^+^CD45^high^ cells (h) and MFI for CD11c expression (i) were comparable between mTNF^wt/wt^ and mTNF^Δ/Δ^ mice. (j) Representative dot blots comparing CD11c expressions on MHCII^+^CD11b^+^CD45^high^ cells in mTNF^wt/wt^ and mTNF^Δ/Δ^ mice 3 days after SCI. (k) Percentages of MHCII^+^, CD11c^+^, and MHCII^+^CD11c^+^ cells in the lesion area. *n* = 4-5 mice/group. (l) Western blot analysis for Iba1 protein expression 3 days after SCI. Data are normalized to *α*-actin protein expression (*n* = 4/group). Results are presented as mean ± SD, ^*∗*^ *p* < 0.05, ^*∗∗*^ *p* < 0.01, Mann–Whitney and Wilcoxon matched-pairs signed rank tests.

In order to further investigate whether genetic ablation of solTNF affected the activation state of microglia and infiltrating cells, we performed flow cytometry and looked for changes in cell populations and MFI of MHCII and CD11c (Figure 5) in the lesion and perilesion areas (proximal and distal to the lesion). The finding of comparable numbers (Figure 5(a)) and percentages (Figure 5(b)) of CD11b^+^CD45^dim^ microglia and CD11b^+^CD45^high^ cells supported the finding that solTNF does not affect microglial and leukocyte recruitment following SCI. In order to investigate whether genetic ablation of solTNF affected activation state, CD11b^+^CD45^dim^ and CD11b^+^CD45^high^ populations were subgated to investigate MHCII (Figures 5(d)–5(f)) and CD11c (Figures 5(g)–5(i)) expression. Neither CD11c nor MHCII was found on the microglia population at this time point (Figures 5(d) and 5(g)). In contrast, MHCII (Figure 5(d)) and CD11c (Figure 5(g)) expression were increased on CD11b^+^CD45^high^ cells with comparable numbers (Figures 5(e) and 5(h)) and expression levels (Figures 5(f) and 5(i)) between genotypes. The number of MHCII^+^ and CD11c^+^ cells and the expression levels were significantly increased in the lesion area compared to the perilesion area. CD11c were only found on MHCII expressing CD11b^+^CD45^high^ cells (Figure 5(j)). In support of these data, the percentages of MHCII^+^, CD11c^+^, and MHCII^+^CD11c^+^ cells in the lesion area were comparable between genotypes (Figure 5(k)). Finally, Iba1 protein expression was investigated 3 days after SCI using Western blotting analysis (Figure 5(l)). However, we found no difference in the expression levels between mTNF^wt/wt^ and mTNF^Δ/Δ^ mice supporting the flow cytometric analysis, demonstrating that genetic ablation of solTNF does not affect the activation state of microglia and infiltrating cells 3 days after SCI.

**Figure 6:**
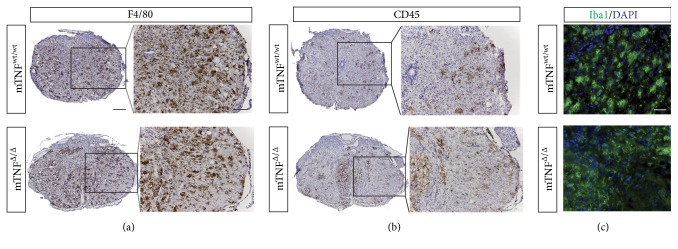
Microglial and leukocyte immunoreactivity 35 days after SCI. (a–c) Immunohistochemical staining for F4/80 (a) and CD45 (b) and immunofluorescent staining for Iba1 (c) were comparable between mTNF^wt/wt^ and mTNF^Δ/Δ^ mice 35 days after SCI. Analysis was based on 5 sections from each animal, *n* = 4 mice/group. Scale bars: (a, b) low magnification = 200 *μ*m and high magnification = 100 *μ*m. (c) 40 *μ*m.

We also evaluated microglia/macrophage activation 35 days after SCI by immunostaining of F4/80, CD45, and Iba1. We observed no difference in the distribution or number of F4/80^+^ (Figure 6(a)), CD45^+^ (Figure 6(b)), or Iba1^+^ (Figure 6(c)) cells between mTNF^wt/wt^ and mTNF^Δ/Δ^ mice.

## 4. Discussion

In the present study, we found that genetic ablation of solTNF, but sustained expression of mTNF, does not affect functional outcome and lesion size after SCI. These findings are in line with previous published papers demonstrating that genetic ablation of TNF [15] and systemic administration of anti-TNF antagonists, such as XPro1595 [16] and etanercept [16, 17], do not affect lesion volume or improve functional outcome after SCI. However, the findings are also in contrast to our own recent studies demonstrating that selective central inhibition of solTNF is neuroprotective, as mice treated epidurally with XPro1595 for 3 consecutive days displayed improved functional outcome, reduced lesion size, and altered neuroinflammation after moderate SCI [16]. As TNF reaches peak levels within the lesioned spinal cord within the first 1-2 hours after SCI [1–4] and elevation of solTNF is a hallmark of acute neuroinflammation (reviewed in [26]) it is possible that, by inhibiting solTNF, using XPro1595 for only 3 consecutive days is neuroprotective, whereas inhibiting solTNF for a sustained period of time (every 3 days for 35 days or as in TNF^−/−^ and mTNF^Δ/Δ^ mice) is not. This is supported by recent hypotheses that under normal physiological conditions solTNF signaling is important for synaptic scaling (reviewed in [26]) and therefore possibly also for neuroregeneration. In the present study, we observed a tendency towards reduced TNFR1 and TNFR2 expression in the spinal cord of mTNF^Δ/Δ^ mice 3 days after SCI, probably as a consequence of reduced solTNF levels. As pharmacological blockage of solTNF, using XPro1595 resulted in sustained TNFR2 and increased TLR4 expression in the lesioned cord, when administered epidurally [16], it is possible that, in order to obtain improved functional recovery and reduced injury following SCI, sustained or increased TNFR2 expression locally is a prerequisite. This is supported by studies in mice with experimental autoimmune encephalomyelitis (EAE), an animal model of multiple sclerosis, where mTNF has been associated with repair and remyelination via oligodendroglial TNFR2 [27], whereas the detrimental effects of solTNF have been associated with TNFR1 signaling [8, 28]. Finally, it is also possible that genetic modifications of TNF expression, as in TNF^−/−^ and mTNF^Δ/Δ^ mice, result in unknown phenotypical modifications of the mice that may not be apparent at first glance. Even though the behavioral phenotype did not appear to be altered in mTNF^Δ/Δ^ mice this is the case in TNF^−/−^ mice [29, 30] and discrepancies in study outcomes between genetically modified TNF models and studies using anti-TNF therapies have been encountered in other CNS models, including models for focal cerebral ischemia and multiple sclerosis (reviewed in [8, 31]). The explanation for these discrepancies so far remains unknown and requires further research into the different functional roles of solTNF and mTNF and the importance of signaling through TNFR1 versus TNFR2.

We also observed a tendency towards a reduction in CCL2 expression in mTNF^Δ/Δ^ mice 3 days after SCI. CCL2 is a monocyte chemotactic protein and an important regulator of macrophage responses [32]. Its expression is upregulated in rat spinal cord tissue within the first 24 hours after SCI [33], as well as in serum samples from SCI patients [34]. Despite our previous findings of a reduced infiltration of monocytes/macrophages into the ischemic brain in our mTNF^Δ/Δ^ mice, we did not observe a difference in infiltrating macrophages 3 days after SCI between mTNF^wt/wt^ and mTNF^Δ/Δ^ mice. We also did not observe any differences in the activation state of microglia or infiltrating cells, as MFI levels for CD11b, CD45, MHCII, and CD11c and Iba1 protein expression were comparable between genotypes 3 days after SCI. Macrophage infiltration and microglial activation are known to peak around day 7 after SCI [35]; hence possible changes in microglia/macrophages are believed to take place at a later time after SCI, which may explain why we in the present study did not observe any change in the number of infiltrating macrophages. The finding that CXCL1, a neutrophil chemoattractant chemokine, did not differ between mTNF^wt/wt^ and mTNF^Δ/Δ^ mice is in line with previous data showing that neutrophils, which are known to infiltrate early (peaking 1 day after injury) [35], were comparable between mTNF^wt/wt^ and mTNF^Δ/Δ^ mice.

The expression of IL-1*β*, a proinflammatory cytokine, increases within the first couple of hours after SCI [4] before the appearance of infiltrating lymphocytes and leukocytes [36], and direct injection of IL-1*β* into the spinal cord enhances vascular permeability and lymphocyte recruitment [37]. In the present study, there was a tendency towards reduced IL-1*β* expression in mTNF^Δ/Δ^ mice 3 days after SCI. In IL-1*α*/*β* ^−/−^ mice, locomotor activity and lesion area improved significantly after SCI, a process believed to be mediated by reduction of inflammatory responses, including a decrease in TNF expression [38]. We have previously shown that IL-1*β* protein levels are decreased in mTNF^Δ/Δ^ mice following experimental stroke, which was accompanied by reduced infarct volumes [12], and that, by increasing the expression of the naturally occurring IL-1 receptor antagonist (IL-1ra) in microglia, infarct volumes following experimental stroke can be reduced [39]. As the neurotoxic effect of IL-1*β*, and hence its deleterious effect on lesion volume, is dependent on the balance between IL-1 and IL-1ra after acute injury to the CNS [39], it is possible that in our conditions a sufficient increase in IL-1ra does not occur, and therefore the decrease in IL-1*β* expression in our mTNF^Δ/Δ^ mice is not sufficient to improve functional recovery and reduce lesion size.

IL-10 is a potent anti-inflammatory cytokine, which has been shown to reduce the development of inflammation and tissue injury associated with SCI [40]. IL-10 has also been shown to reduce IL-1*β* [41] and TNF [42] in rat models of SCI, leading to reduced inflammation, reduced neuronal damage, and improved functional recovery. In our study, we observed a tendency towards reduced IL-10 levels, while TNF and IL-1*β* were decreased, with no improvement in functional outcome 35 days after SCI. As the studies performed by Bethea et al. [42] and Plunkett et al. [41] were in rats and ours was in mice, it is possible that species differences account for the discrepancy. Our studies are in line with the findings by Abraham et al. [43] that showed greater damage at early time points (1 and 7 days) after SCI in IL-10^−/−^ mice but no differences at 14 days after SCI. The pronounced early damage observed in IL-10^−/−^ mice was associated with an almost twofold increase in peripheral neutrophils [43], suggesting an altered innate immune response to injury. Despite the findings of increased IL-10 3 days after SCI, we did not see any difference in infiltrating macrophages and neutrophils. At 35 days after SCI we did not detect differences in CD45^+^, F4/80^+^, or Iba1^+^ microglia/macrophages. However, it should be noted that these observations were based on qualitative immunohistochemistry and further quantitative analysis should be performed in order to conclude whether leukocyte infiltration is affected by genetic ablation of solTNF following SCI.

In summary, our study, using genetically modified mice expressing only the membrane-bound form of TNF, demonstrate that the absence of solTNF does not affect lesion size and functional outcome but suggests that mTNF promotes an anti-inflammatory environment in the lesioned spinal cord that may be more favorable to functional recovery.

## Supplementary Material

Homozygous mTNF^Δ^ ^/Δ^and mTNF^wt^^/^^wt^ littermates were subjected to spinal cord injury and tested in open field test 35 days after injury. Body weights were monitored weekly.
