# Supplementary material for: Genetic Ablation of Soluble TNF Does Not Affect Lesion Size and Functional Recovery after Moderate Spinal Cord Injury in Mice
Source: Mediators Inflamm. 2016 Dec 14;2016:2684098. doi: 10.1155/2016/2684098 (PMC5192339; doi:10.1155/2016/2684098)
Supplement: Supplementary file 1 — Homozygous mTNFΔ /Δand mTNFwt / wt littermates were subjected to spinal cord injury and tested in open field test 35 days after injury. Body weights were monitored weekly. [file 2684098.f1.pdf]

Online supplemental material

Genetic ablation of soluble TNF does not affect lesion size and functional recovery after moderate spinal cord injury in mice

Ditte Gry Ellman[1], Matilda Degn[2§], Minna Christiansen Lund[1§], Bettina Hjelm Clausen[1§], Hans Gram Novrup[1], Simon Bertram Flæng[1], Louise Helskov Jørgensen[2], Lujitha Suntharalingam[1], Åsa Fex Svenningsen[1], Roberta Brambilla[4], Kate Lykke Lambertsen*[1,5,6]

[1]Neurobiology Research, Institute of Molecular Medicine, J.B. Winsloewsvej 21, st, DK-5000 Odense C, Denmark, [2]Rigshospitalet, Department of Diagnostics, Molecular Sleep lab, Nordre Ringvej 69, DK-2600 Glostrup, Denmark, [3]SDU Muscle Research Cluster, Department of Pathology, Institute of Clinical Research, University of Southern Denmark, J.B. Winsloewsvej 15, DK-5000 Odense C, Denmark, [4]The Miami Project to Cure Paralysis, University of Miami Miller School of Medicine, 1095 NW 14th Terrace, Miami, FL 33136, USA, [5]Department of Neurology, Odense University Hospital, Sdr. Boulevard 29, DK-5000 Odense C, Denmark, [6]BRIDGE, Brain Research – Inter-Disciplinary Guided Excellence, Odense Denmark

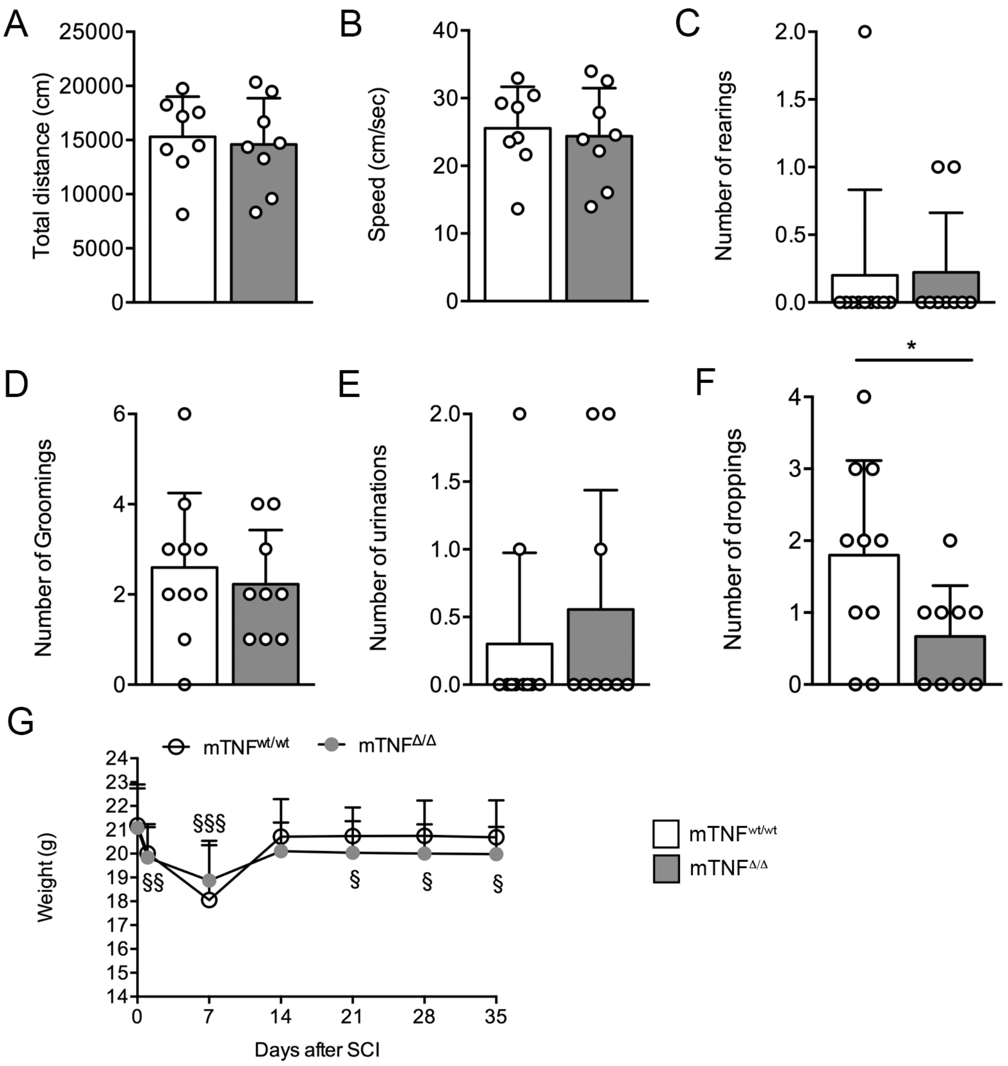

Supplemental Figure 1. Open field test analysis of mTNF$^{wt/wt}$ and mTNF$^{\Delta/\Delta}$ mice 35 days after SCI. (**A-D**) Analysis of locomotor activity in mTNF$^{wt/wt}$ and mTNF$^{\Delta/\Delta}$ mice allowed 35 days survival after SCI showed that both groups of mice travelled a similar distance (**A**), travelled at similar speeds (**B**), an displayed comparable numbers of rearings (**C**) in the open field test. (**D-F**) Analysis of anxiety-related behavior 35 days after SCI demonstrated that the number of groomings and the number of urinations (E) were comparable between genotypes, whereas the total number of droppings were significantly decreased in mTNF$^{\Delta/\Delta}$ compared to in mTNF$^{wt/wt}$ mice (**F**). (**G**) Analysis of weigh change over time after SCI showed that mTNF$^{wt/wt}$ and mTNF$^{\Delta/\Delta}$ mice displayed similar weight drops and weight gain after SCI as compared to baseline (BL). *$p \leq 0.5$ (Mann-Whitney test), §<0.05 compared to BL, §§<0.01

compared to BL §§§<0.001 Compared to BL (two-way RM ANOVA; time ****$p<0.0001$ $F_{6,114=}25.8$, Tukey's post hoc). Data are presented as mean ± SD, n = 8-9 mice/group.
